# Safety limitations of MR‐HIFU treatment near interfaces: a phantom validation

**DOI:** 10.1120/jacmp.v13i2.3739

**Published:** 2012-03-08

**Authors:** Elizabeth Hipp, Ari Partanen, Gregory S. Karczmar, Xiaobing Fan

**Affiliations:** ^1^ Department of Radiology University of Chicago Chicago IL USA; ^2^ Philips Healthcare Cleveland OH USA; ^3^ Department of Physics University of Helsinki Helsinki Finland

**Keywords:** high‐intensity focused ultrasound, temperature elevation, MRI, wave reflection, thermal ablation

## Abstract

Magnetic resonance‐guided high‐intensity focused ultrasound (MR‐HIFU) is a noninvasive image‐guided technique used to thermally ablate solid tumors. During treatment, ultrasound reflections from distal media interfaces can shift prescribed treatment locations. The purpose of this study was to investigate the effect of normal incidence reflections from air, acrylic (modeling bone), and rubber on treatment location, temperature elevation, and heating patterns by performing ultrasound exposures in a tissue‐mimicking phantom and in *ex vivo* porcine tissue using a clinical MR‐HIFU platform. The results demonstrated a shift in treatment location toward the distal interface when targeted closer than 2 cm from the interface, especially for acrylic. Our study demonstrated that the ultrasound wave reflections from a distal air interface had less effect than the acrylic interface (modeling bone) on the heating pattern and focal location. This study provides useful information to better understand the limitations and safety concerns of performing MR‐HIFU treatments with commercial clinical equipment.

PACS numbers: 87.61.‐c, 87.63.D

## I. INTRODUCTION

Magnetic resonance‐guided high‐intensity focused ultrasound (MR‐HIFU) has emerged as a treatment modality for prostate cancer, adenomyoisis, liver tumors, and benign uterine leiomyomas.^(^ ^1^ ^–^ ^4^ ^)^ Treatment of uterine fibroids, in particular, is being accepted by the larger medical community, as MR‐HIFU treatments are now reimbursable by some insurance companies. Understanding the capabilities of commercial systems becomes important for medical physicists asked to implement new treatments using MR‐HIFU.

In much the same way that physicists working with ionizing radiation consider bony and gaseous anatomy in the body differently than soft tissue, these materials must also be treated cautiously when planning MR‐HIFU. Interfaces created by bony anatomy or air at the distal skin surface can cause reflections due to acoustic impedance mismatching. There is concern in the clinical setting that patients' critical structures near target areas may be impacted by reflections from interfaces.

Many investigators have studied ultrasound wave reflection and refraction. Fan and Hynynen^(5)^ used computer simulations and phantom experiments to demonstrate that a tissue interface could cause a shift in the HIFU focal spot location. Using an infrared camera, Zderic et al.^(6)^ demonstrated that thermal effects of HIFU at postfocal tissue–air interfaces could result in significant increases in temperature. By performing computer simulations, as well as experiments, Nell and Myers^(7)^ determined that even if the focal spot is targeted more than 4 cm from bone, heating at the interface may be significant. However, the effect of ultrasound wave reflection from a reflective medium such as bone or air on the treatment target location, on heating pattern, and on temperature elevation has not previously been investigated using a clinical MR‐HIFU system.

In this work, we used a clinical MR‐HIFU platform designed for treatment of uterine fibroids, tissue‐mimicking phantoms, and *ex vivo* tissue to evaluate limitations and safety concerns of wave reflections from a reflective medium. A “treatment cell” — a 4 mm volumetric focal ablation region — was targeted near interfaces at normal incidence to evaluate effects of ultrasound wave reflection. Temperature in that treatment cell region, temperature at the interface, and position of the actual treatment compared to the planned location, were all measured and are reported here.

## II. MATERIALS AND METHODS

### A. Phantom and interface material

Two types of media were used to propagate the ultrasound beam: 1) a partial cone of tissue‐mimicking phantom material (Philips Medical Systems, Vantaa, Finland), ~7cm in the beam propagation direction, and 15 cm and 10 cm in bottom and top diameters, respectively; 2) excised porcine muscle tissue, ~3cm in beam propagation direction and 7cm×12cm in width and length, suspended in a degassed water bath. Three materials were used to create interfaces: air, acrylic, and rubber. Acrylic was used to model bone and rubber is often used as an acoustic dampener. The sizes of the acrylic and rubber pieces were 5cm×5cm square. Some basic acoustic properties of these materials are given in Table 1.

**Table 1:** Acoustic properties for tissue‐mimicking phantom, ex vivo tissue, and interface materials.

*Material*	*Speed of Sound (m/sec)*	*Attenuation (dB/cm MHz)*	*Density (g/cm^3^)*
Phantom^a^	1536	0.417	‐
Muscle tissue^(12)^	1547	1.09	1.05
Air	330	‐	0.00129
Acrylic^(13)^	2870	1.13	1.17
Bone^b^, ^(14)^	3476	6.9	0.400
Rubber^(15)^	1460	0.5–0.7	1.52

a Philips Healthcare.

b Bone is included here for comparison with acrylic.

### B. MR‐HIFU equipment

Experiments were performed using an integrated clinical MR‐HIFU platform (Sonalleve, Philips Medical Systems, Vantaa, Finland and Philips Achieva 1.5T, Philips Healthcare, Best, the Netherlands), which is designed for the ablation of symptomatic uterine fibroids and is capable of sonicating specific target volumes of varying sizes (2–16mm in diameter). A focused ultrasound beam is created using a 256 element array (12 cm radius of curvature, 13 cm aperature) transducer (Imasonic Sa, Besançon, France), operated at 1.2 MHz and propogated out of a sealed degassed water tank and into the target through a thin (50 μm) circular Mylar membrane. For this study, electronic steering the focus in concentric circles was used to create a 4 mm diameter target or “treatment cell”.^(8)^ The system's range for focal position is between ~2.3 and 9.0 cm from the Mylar interface using electronic focusing.

### C. Tissue‐mimicking phantom and ex vivo tissue setup

**Figure 1:**
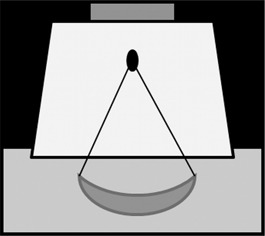
A schematic of the phantom setup showing the transducer, tissue‐mimicking phantom, and interface material. The phantom was placed on the Mylar membrane coupled with a thin layer of degassed water, and the interface reflector material was placed on top of the phantom coupled with ultrasound gel. For studying the phantom‐air interface, the distal phantom surface was left open to the air.

An illustration of the phantom setup is shown in Fig. 1. Interface materials were coupled to the phantom (both tissue‐mimicking and porcine) using standard sonication gel, and an MR sequence was performed to confirm the absence of air bubbles larger than 2 mm at the boundary. The treatment planning software was used to prescribe an ellipsoidal target volume (in‐plane 4 mm short axis, ~1cm beam axis) at three locations: 4, 2, and 1 cm below the interface. While the focal spot size is roughly 2 mm, electronic steering is applied to create a 4 mm diameter elliposoid.^(8)^ Each sonication was 50 W for 20 sec. To mimic soft tissue, *ex vivo* porcine muscle was also used as a propagation media to investigate the effects of the three reflective materials on target locations at depths of 1 and 2 cm below the interface. In this study, reported depths always refer to how far the intended target location was from the interface.

### D. MR imaging protocol for planning and temperature mapping

MR images for treatment planning were acquired using a 3D T2‐weighted turbo spin echo (TSE) pulse sequence (TR/TE=1000/130ms, echo train length=62, field of view (FOV)=250×250×122mm, acquisition matrix=160×111×90, reconstructed in plane voxel size=0.49mm, numberofaverages=2). Dynamic temperature monitoring based on changes in proton resonance frequency (PRF) was performed using 2D fast field echo (FFE) segmented echo‐planar imaging (EPI) (TR/TE=38/20ms, flip $\text{angle}=19.5^{\circ }$, EPI factor=11,FOV=200×200mm, acquisition matrix=100×100, slice thickness=7mm, number of slices=6, in‐plane pixel size=1.25mm, temporal resolution=2.9sec/dynamic). The MR sequence begins first, then after 3 sec, the sonication begins. MR PRF imaging continues during and then after the sonication for an additional 30 sec. The temperature maps were constructed online using the vendor software from phase images using the PRF shift $\left(0.0094\;\text{ppm}{/}^{\circ }C\right)$ temperature measurement technique. The same experiments with the same setup were repeated three times, using a new piece of the *ex vivo* muscle tissue each time. For this study, we waited on average 5 min between sonications, in addition to 30 sec of MR thermometry following the sonication.

### E. Data analysis

The data were analyzed by programs written in‐house in IDL (ITT Visual Information Solutions, Boulder, CO, USA). The intended target location was defined to be the center of the ellipsoidal target volume prescribed during treatment planning. The actual treatment location was determined by finding the maximum temperature voxel within a region of interest (ROI) surrounding the target location, after which the shift from intended location was calculated. ROIs were also drawn at the interface (2 pixels or 2.5 mm deep) to determine the hottest temperature voxel at the interface. Color temperature maps were generated to visually compare effects of different reflective materials.

## III. RESULTS

**Figure 2:**
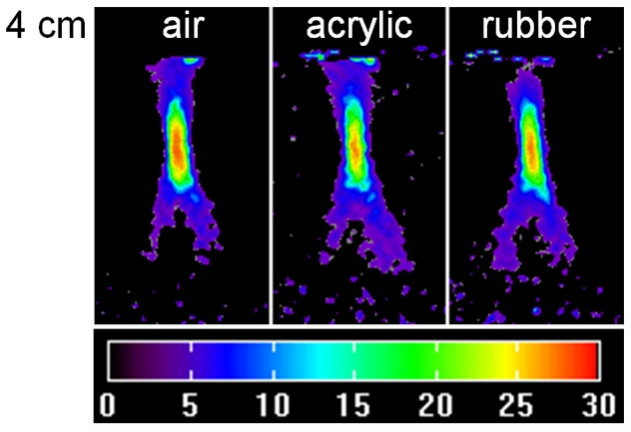
Temperature maps in tissue‐mimicking phantom for sonications targeted at 4 cm below the interface of air, acrylic, and rubber immediately following the sonication. Ultrasound beam propagation direction is from bottom to top. All temperatures (°C) displayed as a relative change from baseline.

For the tissue‐mimicking phantom experiment with sonications targeted to a depth of 4 cm below the distal interface, visual inspection shows the heating patterns at the end of sonication to be similar for all three different interface materials (Fig. 2); they had no measurable effect on the heating pattern at this voxel resolution.

**Figure 3:**
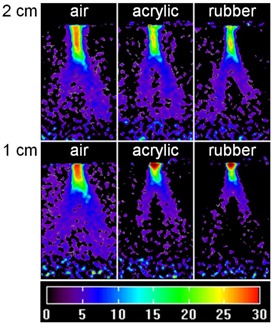
Temperature maps in tissue‐mimicking phantom for sonications targeted at both 2 and 1 cm depth below the interface of air, acrylic, and rubber. Ultrasound beam propagation direction is from bottom to top. All temperatures (°C) displayed as relative change from baseline.

**Figure 4:**
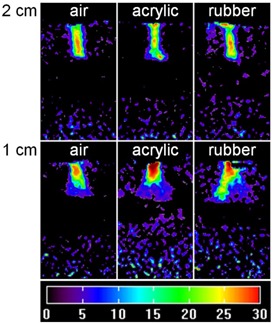
Temperature maps in *ex vivo* muscle tissue for sonications targeted at both 2 and 1 cm depth below the interface of air, acrylic, and rubber. Ultrasound beam propagation direction is from bottom to top. All temperatures (°C) displayed as relative change from baseline.

Figures 3 and 4 show the results for treatment locations prescribed at both 2 and 1 cm below the interface for the tissue‐mimicking phantom and for *ex vivo* tissue, respectively. By qualitative visual inspection, the most ellipsoidal heating pattern for both phantom and *ex vivo* tissue is demonstrated for an air interface, regardless of depth. Interface heating appeared near or at both acrylic and rubber interfaces with both phantom materials at a 1 cm depth.

**Figure 5:**
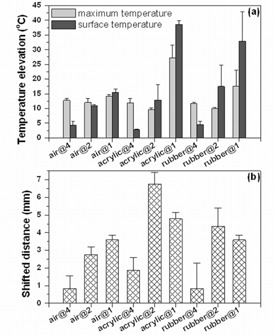
Bar plots displaying: the changes in temperature (a) and shift distance of the target (b) at different depths (4, 2, and 1 cm) of sonication for the tissue‐mimicking phantom with air, acrylic, and rubber interfaces. Maximum temperature elevations from baseline are reported for the center of the targeted heating area (light gray) and at the surface within 2 pixels of the interface (dark gray).

**Figure 6:**
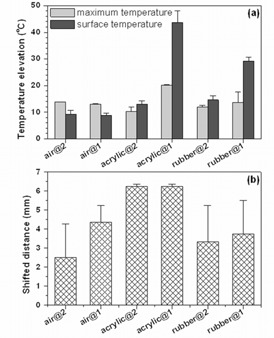
Bar plots displaying: the changes in temperature (a) and shift distance of the target (b) at different depths (2 and 1 cm) of sonication for the *ex vivo* tissue with air, acrylic, and rubber interface. Maximum temperature elevations from baseline are reported for the center of the targeted heating area (light gray) and at the surface within 2 pixels of the interface (dark gray).

The measured maximum temperature at the end of sonication in an ROI of the target volume and in an ROI at the interface, and the calculated shift between the intended and actual treatment locations due to the presence of an interface, are shown in Figs. 5 and 6 for the tissue‐mimicking phantom and *ex vivo* tissue, respectively. Figure 5 shows that for all interface materials, the closer to the interface the target location was, the higher the maximum temperature at the interface. The highest maximum temperature change, an increase of $27.3\pm 4.8^{\circ }C$, was observed at a sonication depth of 1 cm below the acrylic interface (Fig. 5(a)). However, the maximum shift 6.8±0.7mm was observed for the sonication depth of 2 cm below the acrylic interface (Fig. 5(b)). At a sonication depth of 4 cm from the interface, all shifts were within a similar narrow range of ~1–2mm and temperature was increased $\sim 12.0^{\circ }C$ in the target region for all reflective materials. Therefore, the results from the 4 cm depth can be treated as a “no interface” or control case.

Finally, Fig. 6 shows the changes in maximum temperature (a) and shift as a function of sonication depth (b) for *ex vivo* tissue which were similar to that of the tissue‐mimicking phantom. Again, the acrylic tissue interface had the strongest impact on both the maximum temperature and maximum shift.

## IV. DISCUSSION

The effects of ultrasound wave reflection from different interfaces in the tissue‐mimicking phantom and in *ex vivo* tissues were studied using a clinical MR‐HIFU system. The results suggest that at a target depth of 4 cm below an air, acrylic, or rubber interface, the reflections had a minimal impact on the treatment location and that there were no unintentional temperature changes. The target sonications under acrylic experienced greater shifts in position than for air at both the 1 and 2 cm depths. Acrylic and rubber perform similarly as reflectors in terms of changes in temperature elevation at 2 cm for these experimental conditions. However, a statistical test could not be performed due to small sample size. The air reflection showed smaller effects on the heating pattern than acrylic. Still, air interfaces pose an important consideration when treatment planning near the bowels which can contain gas. Therefore, bony anatomy near treatment areas, as well as bowel gas, should be carefully considered when treatment planning. In fact, acoustical impedance mismatches have been taken advantage of to achieve increases in temperatures at bone‐soft tissue interfaces for treatment of metastasis.^(^ ^7^ ^,^ ^9^ ^–^ ^11^ ^)^ One possible solution to avoid unnecessary thermal damage is to use non‐normal incidence where the transducer is positioned at a large incident angle relative to the normal plane of the interface so that any ultrasound that is reflected is outside of the focused treatment area.

Due to limited spatial and temporal MRI resolution, shifts smaller than 1.25 mm may not be accurately reported. Volume averaging effects due to the slice thickness may cause the measured temperatures to be lower than the real temperatures. Here, we are interested in ultrasound wave reflection and the interface heating, so we selected a power and sonication time that are on the lower and shorter end of the spectrum for potential patient treatments. Higher powers and longer sonication times may be needed for well‐perfused areas in patient treatment; the anticipated temperature changes for an increased power can then be expected to be comparable. Nevertheless, our results demonstrate that the heating pattern could be affected, when treatment is targeted 1 or 2 cm from interfaces.

In this study the near field heating was not considered. It is very important that the proximal interface be well‐coupled to the window of the ultrasound transducer, to prevent reflections of the beam from that boundary. Any ultrasound reflected prior to reaching the focus will reduce the power delivered to the treatment cell. Usually, wait time between sonications is higher for volumetric sonications than for sonications into a single focus used here, due to thermal buildup in the near field region.

## V. CONCLUSIONS

Acrylic and rubber perform similarly as reflectors in terms of changes in temperature elevation at 2 cm for these experimental conditions. At a depth of 4 cm, all materials perform similarly without any unintentional heating caused by reflections from the interface.

## ACKNOWLEDGMENTS

This work was supported in part by Philips Healthcare, the Focused Ultrasound Surgery Foundation, and NIH grants R33 (CA100996‐02).

## References

[1] Rabinovici J , Inbar Y , Revel A , et al. Clinical improvement and shrinkage of uterine fibroids after thermal ablation by magnetic resonance‐guided focused ultrasound surgery. Ultrasound Obstet Gynecol. 2007; 30 (5): 771–77. 17899577 10.1002/uog.4099

[2] Siddiqui K , Chopra R , Vedula S , et al. MRI‐guided yransurethral ultrasound therapy of the prostate gland using real‐time thermal mapping: initial studies. Urology. 2010; 76 (6): 1506–11. 20709381 10.1016/j.urology.2010.04.046

[3] Wang W , Wang Y , Tang J . Safety and efficacy of high intensity focused ultrasound ablation therapy for adenomyosis. Acad Radiol. 2009; 16 (11): 1416–23. 19683943 10.1016/j.acra.2009.06.005

[4] Zhang L , Zhu H , Jin C , et al. High‐intensity focused ultrasound (HIFU): effective and safe therapy for hepatocellular carcinoma adjacent to major hepatic veins. Eur Radiol. 2009; 19 (2): 437–45. 18795303 10.1007/s00330-008-1137-0

[5] Fan X and Hynynen K . The effect of wave reflection and refraction at soft‐tissue interfaces during ultrasound hyperthermia treatments. J Acoust Soc Am. 1992; 91 (3): 1727–36. 1564208 10.1121/1.402452

[6] Zderic V , Foley J , Luo W , Vaezy S . Prevention of post‐focal thermal damage by formation of bubbles at the focus during high intensity focused ultrasound therapy. Med Phys. 2008; 35 (10): 4292–99. 18975674 10.1118/1.2975149 PMC2673593

[7] Nell DM and Myers MR . Thermal effects generated by high‐intensity focused ultrasound beams at normal incidence to a bone surface. J Acoust Soc Am. 2010; 127 (1): 549–59. 20059000 10.1121/1.3257547

[8] Kohler MO , Mougenot C , Quesson B , et al. Volumetric HIFU ablation under 3D guidance of rapid MRI thermometry. Med Phys. 2009; 36 (8): 3521–35. 19746786 10.1118/1.3152112

[9] Catane R , Beck A , Inbar Y , et al. MR‐guided focused ultrasound surgery (MRgFUS) for the palliation of pain in patients with bone metastases: preliminary clinical experience. Ann Oncol. 2007; 18 (1): 163–67. 17030549 10.1093/annonc/mdl335

[10] Gianfelice D , Gupta C , Kucharczyk W , Bret P , Havill D , Clemons M . Palliative treatment of painful bone metastases with MR imaging–guided focused ultrasound. Radiology. 2008; 249 (1): 355–63. 18695209 10.1148/radiol.2491071523

[11] Chen W , Zhu H , Zhang L , et al. Primary bone malignancy: effective treatment with high intensity focused ultrasound ablation. Radiology. 2010; 255 (3): 967–78. 20501734 10.1148/radiol.10090374

[12] Mast TD . Empirical relationships between acoustic parameters in human soft tissues. Acoustics Research Letters Online. 2000; 1 (2): 37–42. Available from: http://murphylibrary.uwlax.edu/digital/journals/JASA/JASA2000/pdfs/arlo/vol_1/iss_2/37_1.pdf.

[13] Umchid S . Frequency dependent ultrasonic attenuation coefficient measurement. In: The 3rd International Symposium on Biomedical Engineering. Bangkok, Thailand: ISBME; 2008 p. 234–38.

[14] Hoffmeister BK , Whitten SA , Rho JY . Low‐megahertz ultrasonic properties of bovine cancellous bone. Bone. 2000; 26 (6): 635–42. 10831936 10.1016/s8756-3282(00)00275-1

[15] Browne JE , Ramnarine KV , Watson AJ , Hoskins PR . Assessment of the acoustic properties of common tissue‐mimicking test phantoms. Ultrasound Med Biol. 2003; 29 (7): 1053–60. 12878252 10.1016/s0301-5629(03)00053-x

